# A Mutual Self- and Informant-Report of Cognitive Complaint Correlates with Neuropathological Outcomes in Mild Cognitive Impairment

**DOI:** 10.1371/journal.pone.0141831

**Published:** 2015-11-05

**Authors:** Katherine A. Gifford, Dandan Liu, Timothy J. Hohman, Meng Xu, Xue Han, Raymond R. Romano, Laura R. Fritzsche, Ty Abel, Angela L. Jefferson

**Affiliations:** 1 Vanderbilt Memory & Alzheimer’s Center, Department of Neurology, Vanderbilt University Medical Center, Nashville, Tennessee, United States of America; 2 Department of Biostatistics, Vanderbilt University School of Medicine, Nashville, Tennessee, United States of America; 3 Department of Pathology, Microbiology and Immunology, Vanderbilt University Medical Center, Nashville, Tennessee, United States of America; University of Kentucky, UNITED STATES

## Abstract

**Background:**

This study examines whether different sources of cognitive complaint (i.e., self and informant) predict Alzheimer’s disease (AD) neuropathology in elders with mild cognitive impairment (MCI).

**Methods:**

Data were drawn from the National Alzheimer’s Coordinating Center Uniform and Neuropathology Datasets (observational studies) for participants with a clinical diagnosis of MCI and postmortem examination (n = 1843, 74±8 years, 52% female). Cognitive complaint (0.9±0.5 years prior to autopsy) was classified into four mutually exclusive groups: no complaint, self-only, informant-only, or mutual (both self and informant) complaint. Postmortem neuropathological outcomes included amyloid plaques and neurofibrillary tangles. Proportional odds regression related complaint to neuropathology, adjusting for age, sex, race, education, depressed mood, cognition, APOE4 status, and last clinical visit to death interval.

**Results:**

Mutual complaint related to increased likelihood of meeting NIA/Reagan Institute (OR = 6.58, p = 0.004) and Consortium to Establish a Registry for Alzheimer’s Disease criteria (OR = 5.82, p = 0.03), and increased neurofibrillary tangles (OR = 3.70, p = 0.03), neuritic plaques (OR = 3.52, p = 0.03), and diffuse plaques (OR = 4.35, p = 0.02). Informant-only and self-only complaint was not associated with any neuropathological outcome (all p-values>0.12).

**Conclusions:**

In MCI, mutual cognitive complaint relates to AD pathology whereas self-only or informant-only complaint shows no relation to pathology. Findings support cognitive complaint as a marker of unhealthy brain aging and highlight the importance of obtaining informant corroboration to increase confidence of underlying pathological processes.

## Introduction

Cognitive complaint, or a concern regarding a change in cognition, is a diagnostic criterion for mild cognitive impairment (MCI) [1], a prodromal phase of Alzheimer’s disease (AD), because such complaints purportedly represent a clinically relevant change in cognitive health [2]. In non-demented older adults, a self-reported cognitive complaint predicts cognitive decline [3,4], diagnostic conversion [5,6], smaller medial temporal lobe structures [7], increased in-vivo amyloid burden [8], and post-mortem AD pathology [9–11].

However, a growing body of literature suggests that an informant-reported cognitive complaint or a mutual complaint (a combination of a self and informant complaint) is a better predictor of cognitive decline and diagnostic progression than a self-only complaint, particularly among individuals with MCI [3,12,13]. For example, compared to no complaint, our prior work suggests that an informant-only complaint confers over two times the risk and a mutual complaint confers three times the risk of converting from MCI to dementia [5]. Both informant-only and mutual complaint outperformed a self-only complaint in predicting diagnostic conversion in older adults with MCI [5].

Despite emerging evidence highlighting the importance of informants in assessing cognitive aging, there has been limited empirical consideration of how different sources of complaint relate to AD neuropathological features. In a group of individuals with and without clinical dementia, a self-only complaint was linked to a higher likelihood of meeting AD neuropathological criteria as compared to no complaint [9,10]. Understanding neural changes underlying complaint would enhance the use of complaint as an early marker of disease. It is plausible that complaint may be more strongly associated with neuropathological outcomes when complaint information is pooled from multiple sources than when considered from either source alone [3,5].

The current study relates cognitive complaint captured from participants and informants two years or less prior to the participant’s death to AD neuropathological outcomes in individuals with MCI. Leveraging the National Alzheimer’s Coordinating Center (NACC) database, we hypothesized that individuals with a mutual (both self and informant) complaint have a greater presence of AD neuropathology (i.e., neurofibrillary tangles, plaques) than elders with no cognitive complaint, a self-only complaint, or an informant-only complaint.

## Materials and Methods

### Setting and participants

**Fig 1:**
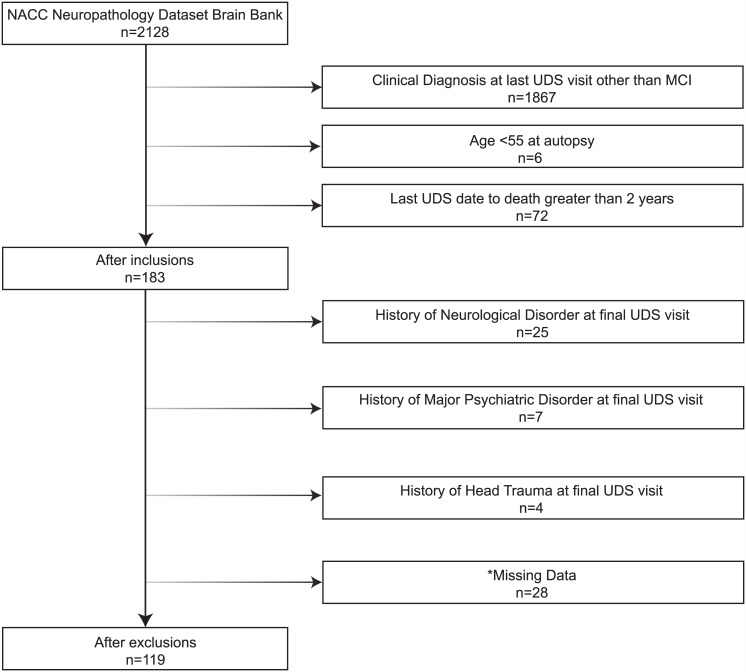
Participant Inclusion & Exclusion Details. Note: The exclusion numbers provided are not mutually exclusive; * = missing data includes demographic and clinical variables (i.e., race, education, depressed mood) and complaint status at last clinical visit.

As previously reported[14], NACC maintains an electronic database of participant information collected from 34 past and current national Alzheimer’s Disease Centers (ADCs) funded through the National Institute on Aging (NIA). For the current analyses, we used data collected from 9/01/2005 to 09/01/2014 as part of the Uniform Data Set (UDS) and the Neuropathology Data Set (NPDS). The UDS includes a standard clinical evaluation protocol, including demographic information, medical history, neurological examination, and neuropsychological evaluation [15]. The NPDS includes a standardized neuropathological examination (Form 9) described below. Participants were 55 years of age and older and diagnosed with MCI at their last UDS visit prior to death, resulting in a sample of 119 participants. See Fig 1 for selection and exclusion details. All participants gave written informed consent at their respective ADC prior to data collection, and the Vanderbilt Institutional Review Board approved secondary analysis of NACC data for this study.

### Cognitive diagnostic classification

The cognitive diagnosis for each NACC participant is based upon clinician judgment or a multi-disciplinary consensus team using information from the comprehensive UDS work-up. MCI determinations are based upon Peterson et al. criteria [16] defined as (a) CDR≤0.5 (reflecting mild severity of impairment), (b) relatively spared activities of daily living, (c) objective cognitive impairment in at least one cognitive domain (i.e., performances falling greater than 1.5 standard deviations outside the age-adjusted normative mean in memory, language, attention, or executive functioning) or a significant decline over time on the neuropsychological evaluation, (d) MMSE score≥23 [17,18], (e) report of a cognitive change by the participant or informant or as observed by a clinician, and (f) absence of a dementing syndrome.

### Cognitive complaint

In the NACC UDS, cognitive complaint is reported by the clinician dichotomously as present or absent for the participant (self) and the informant based on a clinical interview with both parties [3,5]. There are no specific methods for querying cognitive complaint, but examples for capturing such information during clinician-facilitated interviews include asking CDR questions about memory status (i.e., “Do you have problems with your memory or thinking?” or “Does s/he have problems with his/her memory or thinking?”) or general questions about memory and thinking abilities (e.g., “Do you feel that you have been having a significant problem with your short term memory?” or “Are you worried about the participant’s short-term memory?”). Based on the dichotomous classification (present/absent) made by the clinician, four mutually exclusive groups were created for the current study: (1) no complaint (i.e., no self or informant complaint), (2) self-only complaint (but no informant complaint), (3) informant-only complaint (but no self complaint), or (4) mutual complaint (i.e., both a self and informant complaint).

### Neuropathological outcomes

The NACC NPDS protocol has been described previously [19]. Briefly, neurofibrillary tangle densities are evaluated according to published criteria with Braak & Braak stages [20] classified from Stage 0 (no tangles) to Stage VI (most severe with entorhinal, limbic, and neocortical involvement). Diffuse and neuritic plaque presence was rated on a four-level scale, including none, sparse, moderate, and frequent per the NACC NPDS protocol and based upon Consortium to Establish a Registry for Alzheimer’s Disease (CERAD) [21] definition. The likelihood of pathological diagnosis for AD was defined by the NIA/Reagan Institute of the Alzheimer’s Association Consensus Recommendations for the Postmortem Diagnosis of AD (NIA/Reagan criteria) [22] and the CERAD criteria.

### Statistical analysis

Clinical characteristics were based on last UDS visit prior to death and included age, sex, race, education, time from last UDS visit to death, length of time with MCI or cognitive decline (assessed by the age of reported diagnosis or cognitive decline), mood (assessed by the Geriatric Depression Scale [23] total score excluding the question “Do you feel you have more problems with your memory than most?”), global cognition as assessed by the Mini-Mental State Examination (MMSE) [17], Apolipoprotein-E (APOE) genotyping for the ε4 allele (APOE4) status (i.e., positive defined as carrying one or more copies of the ε4 allele or negative defined as carrying no copies of the ε4 allele), MCI subtype classification (i.e., amnestic single domain, amnestic multiple domain, non-amnestic single domain, non-amnestic multiple domain) and primary pathological etiology (when data was available). These clinical characteristics were compared across the four mutually exclusive complaint categories using chi-square and nonparametric analysis of variance tests.

For hypothesis testing, complaint status (using no complaint as the referent) was related to neuropathologic outcomes using proportional odds models for the ordinal outcomes, adjusting for age, sex, education, interval from last clinical visit to death, mood, MMSE, and APOE4 status. We assessed the proportional odds assumption by graphing the mean of each predictor by ordered factor levels against the ordered factor levels to assess possible violation of departure from linearity. In secondary models, to assess the independent relation of complaint to each type of AD pathology, analyses also adjusted for amyloid plaque severity when neurofibrillary tangles were the outcome or neurofibrillary tangle severity when amyloid plaques were an outcome. Second, we repeated all primary models while adjusting for MCI subtype classification (i.e., amnestic single domain, amnestic multiple domain, non-amnestic single domain, non-amnestic multiple domain). Third, we repeated all models covarying for non-AD pathologies (i.e., Lewy Body disease, vascular disease, hippocampal sclerosis). Finally, we repeated all primary models while adjusting for time with MCI or cognitive decline. Significance was set a-priori at p<0.05. Analyses were conducted using *R 3*.*1*.*2* (www.r-project.org).

## Results

### Participant characteristics

A total of 119 participants were included in the study, of which 18 (15%) participants had no complaint, 23 (19%) had a self-only complaint, 19 (16%) had an informant-only complaint, and 59 (50%) participants had a mutual complaint. The four complaint subgroups did not differ on age (p = 0.45), sex (p = 0.37), race (p = 0.52), education (p = 0.05), MMSE (p = 0.24), depressed mood (p = 0.39) or APOE4 status (p = 0.31). Complaint subgroups differed on length of time with cognitive decline or MCI (p = 0.003) with mutual complaint having longer length of cognitive decline than no complaint or self-complaint (p<0.05). Complaint subgroups differed on AD pathological criteria, including NIA/Reagan Institute staging (p = 0.02) with the no complaint group having lower likelihood of meeting criteria than self-only or mutual complaint. The groups differed on the presence of diffuse (p<0.01) and neuritic plaques (p = 0.03) with no complaint having less plaque than mutual complaint and informant-only having less plaque than individuals with mutual complaint. Complaint subgroups did not differ on CERAD criteria (p = 0.15) or Braak staging (p = 0.31). See Table 1.

**Table 1:** Participant Characteristics by Cognitive Complaint Category.

	No Complaint	Self-Only Complaint	Informant-Only Complaint	Mutual Complaint	p-value
Sample size, n	18	23	19	59	
Age at death, mean (SD), y	86.8 (9.3)	90.6 (5.3)	86.7 (10.7)	87.1 (8.3)	0.45
Sex, % female	56	65	53	44	0.37
Race, % White	89	96	100	93	0.52
Education, mean (SD), y	14.6 (2.3)	14.0 (4.2)	16.4 (1.6)	15.6 (3.0)	0.05
MMSE score^†^, mean (SD)	26.5 (3.8)	26.7 (1.8)	25.1 (3.3)	26.3 (2.8)	0.24
Time from last visit to death^‡^, mean (SD), years	0.9 (0.5)	0.9 (0.5)	0.8 (0.5)	0.8 (0.5)	0.97
Length of time with MCI diagnosis, mean (SD), years	2.4 (2.1)	2.7 (2.3)	3.3 (1.5)	4.8 (3.1)	**0.003** *
APOE, % e4 positive	29	33	56	32	0.31
Depression, mean (SD)	3.1 (2.3)	2.5 (1.6)	2.2 (2.5)	3.2 (2.7)	0.39
*MCI diagnosis subtype*					0.25
Amnestic, Single Domain, n (%)	5 (28)	10 (43)	7 (37)	19 (32)	
Amnestic, Multiple Domain, n (%)	6 (33)	7 (30)	10 (53)	26 (44)	
Non-Amnestic, Single Domain, n (%)	6 (33)	2 (9)	2 (11)	10 (17)	
Non-Amnestic, Multiple Domain, n (%)	1 (6)	4 (17)	0 (0)	4 (7)	
*Primary Neuropathological Diagnosis*					
Normal Brain, n (%)	4 (29)	2 (12)	3 (25)	4 (9)	0.24
Alzheimer’s Disease, n (%)	3 (21)	9 (41)	8 (51)	25 (49)	0.29
Vascular Disease, n (%)	2 (15)	4 (22)	2 (14)	7 (16)	0.93
Lewy Body Disease, n (%)	1 (8)	1 (6)	0 (0)	2 (5)	0.81
Frontotemporal Dementia, n (%)	1 (8)	0 (0)	0 (0)	3 (7)	0.51
Hippocampal Sclerosis, n (%)	0 (0)	1 (6)	0 (0)	0 (0)	0.28
*NIA/Reagan Institute*					**0.02** *
High likelihood AD, % yes	6	0	16	24	
Intermediate likelihood AD, % yes	18	59	42	38	
Low likelihood AD, % yes	24	27	26	16	
Criteria not met, % yes	53	14	16	22	
*CERAD Criteria*					0.15
Definite AD (%)	8	0	12	27	
Probable AD (%)	0	17	25	25	
Possible AD (%)	23	25	25	11	
Criteria not met (%)	69	58	38	36	
*Braak Stages*					0.31
Stage 0	6	0	0	2	
Stage I	24	5	11	3	
Stage II	29	23	16	17	
Stage III	18	32	21	22	
Stage IV	18	32	32	33	
Stage V	0	9	5	16	
Stage VI	6	0	16	7	
*Neuritic plaques*					**0.03** *
None	50	14	21	24	
Sparse	28	36	47	15	
Moderate	17	32	16	39	
Frequent	6	18	16	22	
*Diffuse plaques*					**<0.01** *
None	40	16	18	13	
Sparse	40	42	47	15	
Moderate	7	11	0	34	
Frequent	13	32	35	38	

*Note*: Complaint categories are mutually exclusive and defined based on clinician-facilitated interviews with the participant and informant; depression assessed by the Geriatric Depression Scale excluding the cognitive complaint question; MMSE = Mini-Mental State Examination;

*p<0.05;

^†^MMSE score range from 0–30 with lower score = worse performance;

^‡^Follow-up period is time from the last clinic visit to death.

### Cognitive complaint & neuropathological measures

Compared to participants with no complaint, participants with a mutual complaint had a higher likelihood of meeting NIA/Reagan criteria (OR = 6.58, p = 0.004). No differences were detected between no complaint and self-only or informant-only complaint groups (p>0.17; see Table 2).

**Table 2:** Odds Ratios for Neuropathology Outcomes in MCI.

	Self-Only Complaint^ŧ^			Informant-Only Complaint^ŧ^			Mutual Complaint^ŧ^		
	OR	95% CI	p-value	OR	95% CI	p-value	OR	95% CI	p-value
NIA/Reagan Criteria	2.57	0.67, 9.91	0.17	3.05	0.62, 14.96	0.17	6.58	1.82, 23.77	**0.004** *
CERAD Criteria	0.67	0.09, 4.90	0.70	2.87	0.31, 26.56	0.35	5.82	1.19, 28.49	**0.03** *
Braak Stages	1.79	0.50, 6.39	0.37	2.27	0.51, 10.17	0.29	3.70	1.15, 11.92	**0.03** *
Neuritic Plaques	2.83	0.76, 10.48	0.12	1.20	0.27, 5.39	0.81	3.52	1.08, 11.55	**0.03** *
Diffuse Plaques	2.46	0.61, 10.00	0.21	1.92	0.40, 9.23	0.41	4.35	1.23, 15.43	**0.02** *

*Note*: Complaint categories are mutually exclusive and defined based on clinician-facilitated interviews with the participant and informant; CI = confidence interval;

* = p < .05;

^**ŧ**^predictor referent = no complaint; proportional odds models adjusted for age, sex, race, education, global cognition, depressed mood, APOE4 carrier status, and time from last clinic visit to death.

Compared to participants with no complaint, participants with a mutual complaint were more likely to have a higher Braak stage (OR = 3.70, p = 0.03). No differences were detected between no complaint and self-only or informant-only complaint groups (p> 0.29; see Table 2).

Compared to participants with no complaint, participants with a mutual complaint were more likely to meet CERAD criteria for AD (OR = 5.82, p = 0.03). No differences were detected between the no complaint group and self-only or informant-only complaint groups (all p-values>0.35; see Table 2).

Compared to participants with no complaint, participants with a mutual complaint were more likely to have neuritic (OR = 3.52, p = 0.03) and diffuse plaques (OR = 4.35, p = 0.02). No differences were detected between the no complaint group and self-only or informant-only complaint groups for neuritic (p>0.12) or diffuse plaques (p>0.21; see Table 2). For all analyses, assessment of the proportional odds assumption revealed no departure from linearity for any predictor or outcome combination (data not shown).

### Secondary analysis

When Braak severity was included as a covariate, individuals with a mutual complaint evidenced greater likelihood of meeting CERAD criteria for AD (OR = 4.91, p = 0.03) and greater neuritic (OR = 3.71, p = 0.02) and diffuse plaque frequency (OR = 5.43, p = 0.005) than individuals with no complaint. Compared to the no complaint group, no differences were detected in the self-only or informant-only complaint groups (all p-values>0.05). When CERAD criteria was used as a covariate, a mutual complaint was related to greater Braak severity than no complaint (OR = 7.09, p = 0.005), but no differences were detected in the self-only or informant-only complaint groups compared to the no complaint group (all p-values>0.06). Primary results were unchanged when the models were covaried for MCI diagnosis status (i.e., amnestic single domain, amnestic multiple domain, non-amnestic single domain, non-amnestic multiple domain), or non-AD pathologies (i.e., Lewy Body disease, vascular disease, hippocampal sclerosis; data not shown). When time with MCI/cognitive decline was added as a covariate, all significant results were rendered null (p>0.05; data not shown).

## Discussion

Leveraging a large, multicenter cohort, we found that among individuals with a diagnosis of MCI prior to death, a mutual (both self and informant) cognitive complaint was associated with a greater likelihood of having a pathological diagnosis of AD, more frequent neuritic and diffuse plaques, and more frequent neurofibrillary tangles. In contrast, a self-only complaint or informant-only complaint yielded no associations with AD pathologic burden. Taken cumulatively, these results highlight that the presence of a mutual complaint may be an indicator of underlying AD neuropathology early in the disease course.

Our findings not only align with previous reports that a cognitive complaint is related to AD pathology [9–11] in non-demented older adults, but they also provide novel information about cognitive complaint in cognitive aging. First, our study is among the first to highlight possible biological underpinnings of cognitive complaint in MCI, offering additional support for the use of cognitive complaint as a potential marker of unhealthy brain aging. Although there is a growing body of literature suggesting that cognitive complaint carries substantial predictive power for future decline in cognitively normal older adults [3,24], complaints may also offer important information about the presence of AD neuropathology in the earliest clinical stages of the disease (i.e., MCI). This finding is consistent with in-vivo studies of amyloid binding in the brain [8,25] and other post-mortem studies [11] suggesting a relation between cognitive complaint and amyloid deposition. Assessment of complaints across the cognitive aging spectrum may be an important enhancement to the clinical evaluation of older adults and prove informative in shaping future intervention strategies.

Second, the current study presents some of the first data examining how different sources of cognitive complaint (i.e., self-only, informant-only, mutual) relate to AD pathology. These results underscore the need to consider the source of complaints in the context of clinical diagnosis, which supports previous work demonstrating the value of complaint source in normal cognition and MCI populations. That is, previous studies have shown that a mutual complaint is more strongly correlated with diagnostic conversion and cognitive decline when compared to one source of complaint alone [3,5]. Combined with prior data, the current results suggest that a mutual complaint (among all complaint categories) may be a stronger predictor of unhealthy brain aging, specifically AD pathology.

The minimal association between either a self-only complaint or an informant-only complaint and increased risk for AD pathology was unexpected. These null observations were particularly surprising given previous reports in non-demented cohorts linking a self-only complaint with increased AD pathologic burden [9] and data suggesting that informant report is superior to self-report [5,12]. One explanation is that the current study was underpowered to detect associations between self-only and informant-only complaint groups given the absence of continuous pathology measures available for analysis and relatively small sample size in the self-only (n = 23) and informant-only complaint groups (n = 19). Another surprising finding was the null results when time with MCI/cognitive decline was included as a covariate in primary models. Although time with MCI/cognitive decline was not related to neuropathology, it was related to the type of cognitive complaint (i.e., longer time was related to the presence of mutual complaint). One reason for the null results could be that the current sample was underpowered to examine this association given the relatively small sample size in many groups (i.e., n = 4 with no complaint and frequent/moderate neuritic plaques). Further research using an enhanced sample is needed to detect these associations.

The current study has a number of strengths. First, as compared to previous research considering self-only complaint, the methodology incorporated a combination of complaint sources. Second, the focus on MCI greatly enhances the literature by providing clinically meaningful information at a time when older adults are showing the earliest clinical manifestations of cognitive decline. Individuals often present to their healthcare providers due to noticeable but minimal changes in cognition and daily functioning during this prodromal phase of AD, making it a critical period for clinicians to evaluate, intervene, and treat this population most effectively. The current findings highlight the relevance of attending to cognitive complaint, particularly in the presence of a mutual complaint, at early stages of cognitive impairment. The use of NACC data represents a number of strengths and enhancements to previous literature, including standardized clinical diagnostic criteria, standardized neuropathological criteria, and a sample that mimics memory clinic patient referrals where the relation between complaints and neuropathology may be most relevant to intervention.

Despite numerous strengths, several key limitations must be considered. NACC participants, although representative of 34 past and present ADCs throughout the United States, are primarily White and well-educated. These participants are often recruited from a memory clinic or self-referred for participation. Such factors may lead to different baseline characteristics as compared to a population-based study, limiting the generalizability of our results to the community. However, the sample may provide clinically relevant information specific to healthcare providers. Second, methods for determining cognitive complaints vary across sites and are rated in a dichotomous nature, precluding assessment of complaint gradations or severity in relation to neuropathology outcomes. Nevertheless, the simplicity of the dichotomous rating may have more relevance for active primary care settings where an extensive cognitive complaint interview would be impractical.

## Conclusions

The current study highlights the possible association between a mutual (i.e., self- and informant-report) cognitive complaint with AD pathology in older adults with MCI. These findings are one important step in understanding how the source of cognitive complaint during clinical evaluation may relate to neuropathological features of AD. Further research is needed to better understand how cognitive complaint relates to neuropathological markers in normal cognition prior to the onset of clinical symptoms, how the association between complaint and AD pathology changes over the course of the disease, and how different methods of assessing complaint correspond to future pathological changes.
